# Castration-resistant prostate cancer cells are dependent on the high activity of CDK7

**DOI:** 10.1007/s00432-022-04475-3

**Published:** 2022-11-18

**Authors:** Satu Pallasaho, Aishwarya Gondane, Anni Kuivalainen, Samuel Girmay, Siver Moestue, Massimo Loda, Harri M. Itkonen

**Affiliations:** 1 Department of Biochemistry and Developmental Biology, Faculty of Medicine, University of Helsinki, PL 63 (Haartmaninkatu 8), 00014 Helsinki, Finland; 2 Department of Clinical and Molecular Medicine, NTNU, Trondheim, Norway; 3 Department of Pharmacy, Nord University, Namsos, Bodø, Norway; 4 Department of Pathology and Laboratory Medicine, Weill Cornell Medicine, New York-Presbyterian Hospital, New York, NY USA; 5 The Broad Institute of Harvard and MIT, Cambridge, MA USA; 6 The New York Genome Center, New York, NY USA

**Keywords:** Cyclin-dependent kinase 7, Castration-resistant prostate cancer, O-GlcNAc transferase, Cyclin-dependent kinase 9

## Abstract

**Purpose:**

Prostate cancer (PC) is successfully treated with anti-androgens; however, a significant proportion of patients develop resistance against this therapy. Anti-androgen-resistant disease (castration-resistant prostate cancer; CRPC) is currently incurable. Cyclin-dependent kinase 7 (CDK7) is positioned to positively regulate both cell cycle and transcription, the two features critical for the rapid proliferation of the CRPC cells. Here, we assess if CDK7 is a viable target to halt the proliferation of CRPC cells.

**Methods:**

We use recently developed clinically relevant compounds targeting CDK7 and multiple cell proliferation assays to probe the importance of this kinase for the proliferation of normal, androgen-dependent, and CRPC cells. PC patient data were used to evaluate expression of CDK7 at different disease-stages. Finally, comprehensive glycoproteome-profiling was performed to evaluate CDK7 inhibitor effects on androgen-dependent and CRPC cells.

**Results:**

We show that CDK7 is overexpressed in PC patients with poor prognosis, and that CRPC cells are highly sensitive to compounds targeting CDK7. Inhibition of O-GlcNAc transferase sensitizes the CRPC, but not androgen-dependent PC cells, to CDK7 inhibitors. Glycoproteome-profiling revealed that CDK7 inhibition induces hyper-O-GlcNAcylation of the positive transcription elongation complex (pTEFB: CDK9 and CCNT1) in the CRPC cells. Accordingly, co-targeting of CDK7 and CDK9 synergistically blocks the proliferation of the CRPC cells but does not have anti-proliferative effects in the normal prostate cells.

**Conclusion:**

We show that CRPC cells, but not normal prostate cells, are addicted on the high activity of the key transcriptional kinases, CDK7 and CDK9.

**Supplementary Information:**

The online version contains supplementary material available at 10.1007/s00432-022-04475-3.

## Introduction

Prostate cancer is the most common cancer and the second leading cause of cancer-associated deaths in men (Siegel et al. 2021). The growth of prostate cancer cells is driven by the hyper-activated transcription factor, androgen receptor (AR) (Mills 2014). Prevention of AR activity halts the disease progression, and prostate cancer is successfully treated with androgen-deprivation therapy (Sandhu et al. 2021). However, a significant proportion of the patients develop castration-resistant prostate cancer (CRPC). CRPC cells do not respond to androgen-deprivation therapy, and at this point, the disease is currently incurable.

Proliferation of both the prostate cancer and the CRPC cells depends on the rapid progression through cell cycle and on the high activity of the transcriptional machinery. Cyclin-dependent kinase 7 (CDK7) is positioned to positively regulate both. CDK7 promotes transcription by directly phosphorylating the carboxyl-terminal domain (CTD) of RNA polymerase II (RNA Pol II) to promote transcription initiation (Chou et al. 2020; Fisher 2019). In addition, CDK7-dependent phosphorylation of the cell-cycle CDKs promotes cell-cycle progression (Diab et al. 2020). As a positive regulator of both the transcription and the cell cycle, CDK7 is an attractive target for cancer therapeutics.

Previously, CDK7 has been proposed to be an important regulator of the AR-dependent transcription in CRPC cells (Rasool et al. 2019). These discoveries were made using compounds that target both CDK7 and CDK12 (Olson et al. 2019). Development of compounds that specifically target only CDK7, YKL-5-124 (Olson et al. 2019), and Samuraciclib (Patel et al. 2018) enables the assessment of CDK7’s role in CRPC cells.

Here, we show that CDK7 inhibition is toxic to CRPC cells but not to normal prostate cells. The CRPC-selective effects of CDK7 inhibitors can be enhanced by co-targeting O-GlcNAc transferase (OGT). OGT O-GlcNAcylates thousands of proteins, and we use comprehensive glycoproteomic profiling to understand how depletion of CDK7 activity remodels OGT substrate repertoire in the CRPC cells and in the androgen-dependent prostate cancer cells. These experiments revealed that transcription elongation kinase CDK9 is increasingly O-GlcNAcylated only in the CRPC cells in response to CDK7 inhibition. Finally, combined inhibition of CDK7 and CDK9 induces synergistic anti-proliferative effects against CRPC cells but is not toxic to normal prostate cells.

## Materials and methods

### Cell culture and compounds

Cell lines 22RV1, C4-2, LNCaP, PC3, and RWPE-1 were obtained from the American Tissue Culture Collection. PNT1 cells were acquired from Sigma. LNCaP95 (LN95) cell line was kindly provided by Professor Stephen Plymate (University of Washington). 22RV1, C4-2, LNCaP, and PNT1 cells were maintained in the RPMI medium supplemented with 10% fetal bovine serum (FBS). LN95 and RWPE-1 cells were maintained in 10% charcoal-stripped FBS in the phenol red-free RPMI media and in the keratinocyte serum-free media, respectively. Samuraciclib (Patel et al. 2018), YKL-5-124 (Olson et al. 2019), OSMI-4 (Martin et al. 2018), NVP2 (Olson et al. 2018), and Thiamet G (Yuzwa et al. 2008) were obtained from MedChemExpress.

### Proliferation and viability assays

Proliferation rate of the cells was measured using Incucyte live-cell imaging system according to manufacturer’s (Sartorius) instructions. Cell viability was measured using CellTiter-Glo^®^ and CellTiter-Glo^®^ 2.0 assays (Promega). Synergy calculations were performed using the tool reported by Ianevski et al. (2020). For detection of cell death activation, we used IncuCyte Caspase-3/7 Green Reagent for apoptosis (Sartorius). Colony formation was assessed using crystal violet staining as previously described (Barkovskaya et al. 2020) with minor changes. Briefly, cells growing in a 6-well plate were washed with PBS, fixed with ice-cold 70% methanol for 2 min, and then with 100% methanol for 10 min. After cells dried out, they were stained with 0.05% crystal violet in 25% methanol for 10 min and washed twice with deionized water. Cells were allowed to dry after which 300 µL of 10% acetic acid was added to each well to extract the dye, and the absorbance was measured at 590 nm. Boxplots were made using R version 4.1.1 in R studio 2021.09.0.

### O-GlcNAc immunoprecipitation and mass spectrometry

O-GlcNAc immunoprecipitation was performed using Pierce Direct IP Kit (ThermoFisher Scientific) according to the manufacturer’s protocol and combination of two antibodies: RL2 (Abcam: ab2739) and CTD110.6 (Cell Signaling Technologies: 9875). Cell lysis buffer was supplemented with protease and OGA inhibitors (latter: Thiamet G). Mass spectrometry analysis of biological triplicate samples was performed by the Weill Cornell Medicine (WCM) Meyer Cancer Center Proteomics & Metabolomics Core Facility.

### NMR-based metabolite profiling

Cell extract preparation was performed at + 4 °C. First, cells were washed with PBS, after which they were vortexed in 80% ethanol and centrifuged (4000 rpm, 5 min). The supernatant was collected and the vortexing in 80% ethanol was repeated for the pellet. Next, the supernatant samples were dried in SpeedVac (Savant SVC-100, Thermo Fisher Scientific), stored at − 80 °C, and reconstituted in 600 µL D_2_O immediately before NMR analysis. The samples were transferred to 5 mm NMR tubes (SampleJet, Bruker Biospin GmbH), and analyzed on a 600 MHz Bruker Avance III NMR spectrometer (Bruker Biospin GmbH) with a 5 mm QCI Cryoprobe. Proton spectra were acquired at 300 K using 1D NOESY (Bruker: noesygppr1d) with presaturation and spoiler gradients as described previously (Itkonen et al. 2016). Data were collected with 32 scans and four dummy scans, Fourier transformed with an exponential line broadening of 0.3 Hz, baseline corrected using asymmetric least-squares method (Eilers 2004), and peak aligned using icoshift (Savorani et al. 2010). The water resonance and areas in the spectra with contamination and noise only were removed. All spectra were mean normalized and mean centered before principal component analysis was performed with PLS toolbox v8.2.1 (Eigenvector Research).

## Results

### Prostate cancer cells overexpress CDK7 and are sensitive to compounds targeting CDK7

**Fig. 1:**
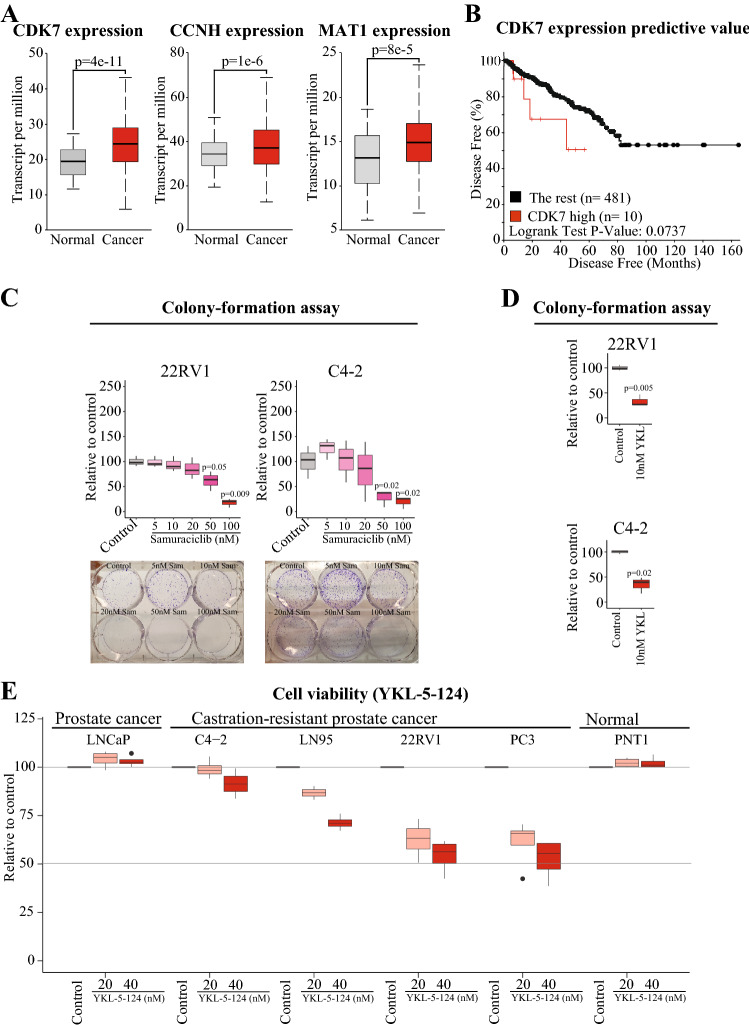
Castration-resistant prostate cancer cells are dependent on the high CDK7 activity. **A** Evaluation of CDK7, Cyclin H (CCNH), and MAT1 expression in normal and prostate cancer tissue. The figure was generated using the Ualcan-portal (Chandrashekar et al. 2017) and the TCGA prostate cancer data set. **B** Increased expression of CDK7 is associated with disease recurrence. The plot was generated using the TCGA prostate cancer dataset accessed through the cBioportal (Cerami et al. 2012; Gao et al. 2013). **C**, **D** Colony-formation assay after 7 days. Boxplots present the data of at least three biological replicates. Paired two-tailed Student’s *t* test was used to evaluate the significance. **E** Viability measurement of cells after 4 days of treatment. Boxplots presenting four biological replicates; control sample was always set to 100 and treatments were normalized to this

We hypothesized that the aggressive prostate cancer cells would upregulate CDK7 and its positive regulators, because this kinase is involved in two processes essential for the rapid proliferation: transcription and the cell cycle. To test this, we used the TCGA Prostate Adenocarcinoma-dataset. Indeed, CDK7 expression was significantly higher in the prostate cancer tissue when compared to the normal tissue (Fig. 1A). CDK7 is positively regulated through complex formation with Cyclin H and MAT1 (Fisher 2019), and also, these two were significantly overexpressed in prostate cancer tissue when compared to normal samples (Fig. 1A). In addition, increased expression of CDK7 was associated with the rapid disease recurrence (Fig. 1B). These data are in accordance with a previous report showing overexpression of CDK7 in prostate cancer (Paulsen et al. 2022). Here, we show that also the binding partners of CDK7 are overexpressed in prostate cancer.

Overexpression of CDK7 and its positive regulators implies that increased activity of this kinase is beneficial for prostate cancer cells. We used two structurally unrelated CDK7 inhibitors, Samuraciclib (Patel et al. 2018) and YKL-5-124 (Olson et al. 2019), to probe the importance of the high CDK7 activity for the survival of CRPC cells. Samuraciclib significantly inhibited the colony-formation ability of the two CRPC models (C4-2 and 22RV1) (Fig. 1C). Similarly, YKL-5-124 inhibited the colony-formation ability of the CRPC cells already at 10 nM dose (Fig. 1D). In addition, inhibition of CDK7 using YKL-5-124 dose-dependently reduced the viability of the CRPC cells (Fig. 1E). Interestingly, CDK7 inhibition did not affect the viability of the androgen-dependent LNCaP cell line or the normal prostate cells (PNT1; Fig. 1E).

To summarize our findings so far, CDK7 and its positive regulators are overexpressed in the tumors of prostate cancer patients with poor prognosis, and inhibition of CDK7 activity selectively halts the proliferation of the CRPC cells. The decline in cell proliferation was significant but still a high number of the CRPC cells survived the treatment. We therefore reasoned that the right combinatorial treatment strategy could enhance the CRPC-selective anti-proliferative effects of CDK7 inhibitors.

### Co-targeting of OGT and CDK7 causes CRPC-selective anti-proliferative effects

**Fig. 2:**
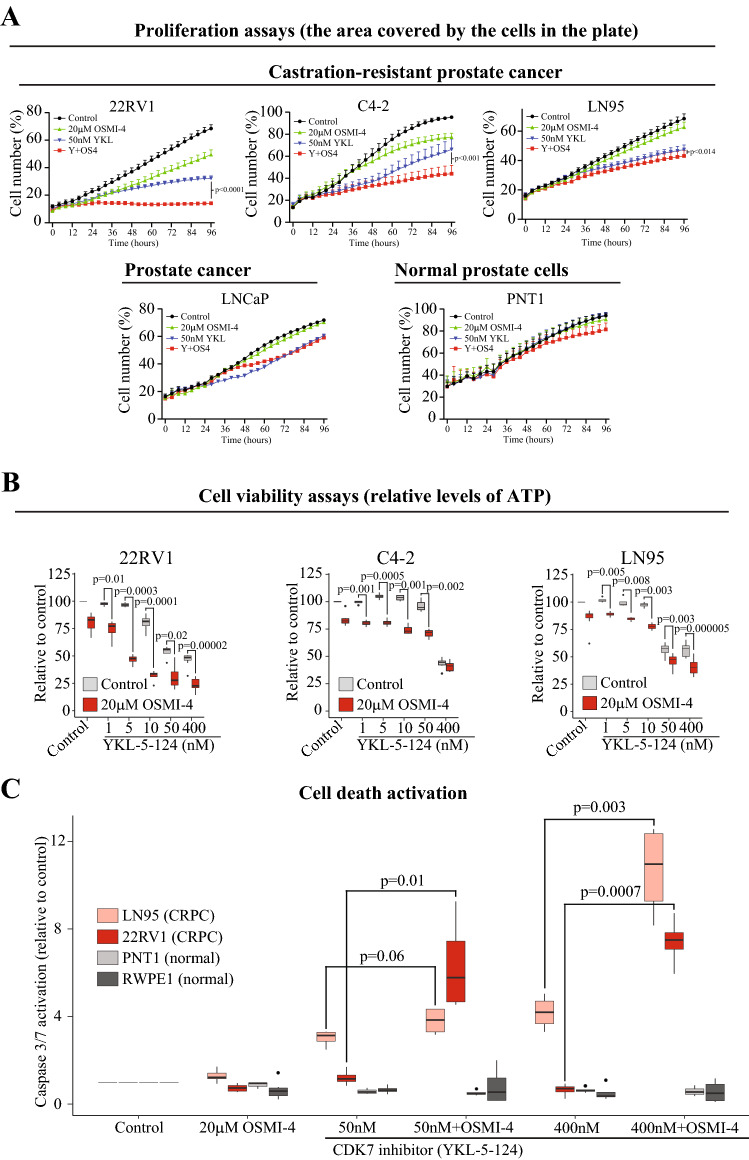
Co-targeting of OGT and CDK7 induces CRPC-selective growth defects. Data of four biological replicates in A, B, and C, and two-tailed paired Student’s *t* test was used to evaluate the significance in each case. **A** Proliferation rate of the indicated cell lines was followed over 4 days using live-cell imaging. **B** Cells were treated as indicated for 4 days and viability was assessed. **C** Activation of caspases 3/7 after 4 days of the indicated treatments

We hypothesized that depletion of O-GlcNAc transferase (OGT) activity might enhance the efficacy of CDK7 inhibitors. OGT is overexpressed in prostate cancer patients (Itkonen and Mills 2013), and the enzyme is enriched in the transcriptionally active chromatin in the prostate cancer cells (Itkonen et al. 2019). Interestingly, depletion of OGT activity using OSMI-4 (Martin et al. 2018) significantly enhanced the efficacy of CDK7 inhibitor YKL-5-124 in three models of CRPC but not in the androgen-dependent LNCaP cell line or the normal prostate cells (Fig. 2A). Cell viability assays demonstrated highly significant combinatorial toxicity of OGT and CDK7 inhibition on three CRPC models (Fig. 2B). We confirmed the combinatorial effects on viability using another CDK7 inhibitor, Samuraciclib (Suppl. Figure 1). Finally, we used caspase-activation assays to establish if the robust anti-proliferative effects of co-targeting OGT and CDK7 are explained in part through the cell death activation. Depletion of OGT activity significantly enhanced the CDK7 inhibitor-induced cell death response in the CRPC cells but had no effect on the normal prostate cells (Fig. 2C).

These data show that the CRPC cells are addicted on the high activity of both CDK7 and OGT. Interestingly, these combinatorial effects are seen only in the CRPC cells and not in the androgen-dependent prostate cancer cells or in the normal prostate cells. By understanding how OGT activity is remodeled in the CRPC cells in response to CDK7 inhibition, we may be able to design a CRPC-selective combinatorial treatment strategy.

### CDK7 inhibition selectively remodels OGT activity in the CRPC cells

**Fig. 3:**
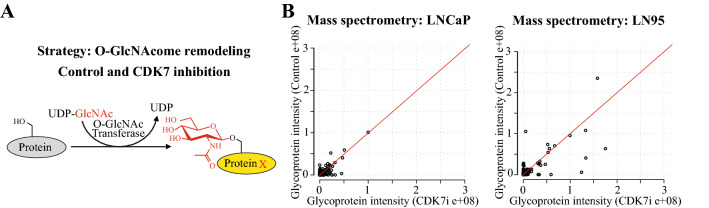
Characterization of CDK7 inhibitor-induced O-GlcNAcome in prostate cancer and CRPC cells. **A** We hypothesized that OGT substrate-selectivity is altered when CDK7 is inhibited. To test this, we treated LNCaP (prostate cancer) and LN95 (CRPC) cells with CDK7 inhibitor, and used mass spectrometry to identify the proteins that are O-GlcNAcylated in each model (denoted as protein X). **B** Scatter plots showing the O-GlcNAcylated proteins in basal conditions and in response to 4 h treatment with 500 nM YKL-5-124 as identified using mass spectrometry. Data shown are an average of three biological replicates in each condition. The signal intensity values represented here were normalized to IgG

OGT activity is remodeled in response to stress (Gondane et al. 2022b; Martinez et al. 2017), and we hypothesized that its activity is selectively remodeled in CRPC cells when CDK7 is inhibited. Proteins selectively hyper-O-GlcNAcylated in response to CDK7 inhibition represent candidates, which can be targeted to replicate the combinatorial lethal effects of OGT and CDK7 inhibitors. We used an isogenic model system and mass spectrometry to probe if OGT’s substrate repertoire in response to CDK7 inhibition differs between the androgen-dependent and CRPC cells (Fig. 3A). LNCaP and LN95 isogenic cell line pair models the development of the CRPC: LNCaP cells depend on androgens to proliferate, and LN95 cell line was established via an extended androgen depletion of the LNCaP cells. LN95 cells proliferate in the absence of androgens.

We performed biological triplicate O-GlcNAc immunoprecipitation (IP) experiments in LNCaP and LN95 cells after short-term CDK7 inhibition (4 h) and analyzed the IPs using mass spectrometry. Interestingly, LN95 cells had twice more proteins whose O-GlcNAcylation increased by at least 20% in all three biological replicate experiments, when compared to LNCaP cells (LNCaP: 85 proteins and LN95: 188 proteins, Fig. 3B and Suppl. Figure 2A). Pathway enrichment analysis of the affected proteins identified metabolic processes; however, we did not detect any robust changes in the metabolite profiles of CRPC or normal cells after CDK7 inhibitor treatment (Suppl. Figure 2B and C).

Based on these data, CDK7 inhibition induces robust changes in the OGT’s substrate repertoire in CRPC cells. We hypothesize that some of the proteins increasingly O-GlcNAcylated when CDK7 activity is depleted form combinatorial lethal pairs with CDK7 inhibitor against the CRPC cells.

### Combined targeting of CDK7 and CDK9 selectively blocks proliferation of prostate cancer cells

We set out to identify proteins that could be directly targeted using specific compounds to replace OGT inhibitor in combinatorial lethality between CDK7 and OGT inhibitors. We noted that proteins directly linked to RNA Pol II pause-release after transcription initiation were increasingly O-GlcNAcylated only in the CRPC cells in response to CDK7 inhibition (Suppl. Table 1). These included SPT5 Homolog, DSIF Elongation Factor Subunit (SUPT5H), CDK9, and Cyclin T1 (CCNT1). Previously, we reported that inhibition of other transcriptional kinases CDK9 and CDK12/13 also increases O-GlcNAcylation of CDK9 (Gondane et al. 2022a, b). SUPT5H is part of the complex promoting the pause of RNA Pol II at sites proximal to the promoter, while CDK9-CCNT1 forms the P-TEFB complex, a protein machinery required for the release of RNA Pol II for transcription elongation (Chou et al. 2020).

**Fig. 4:**
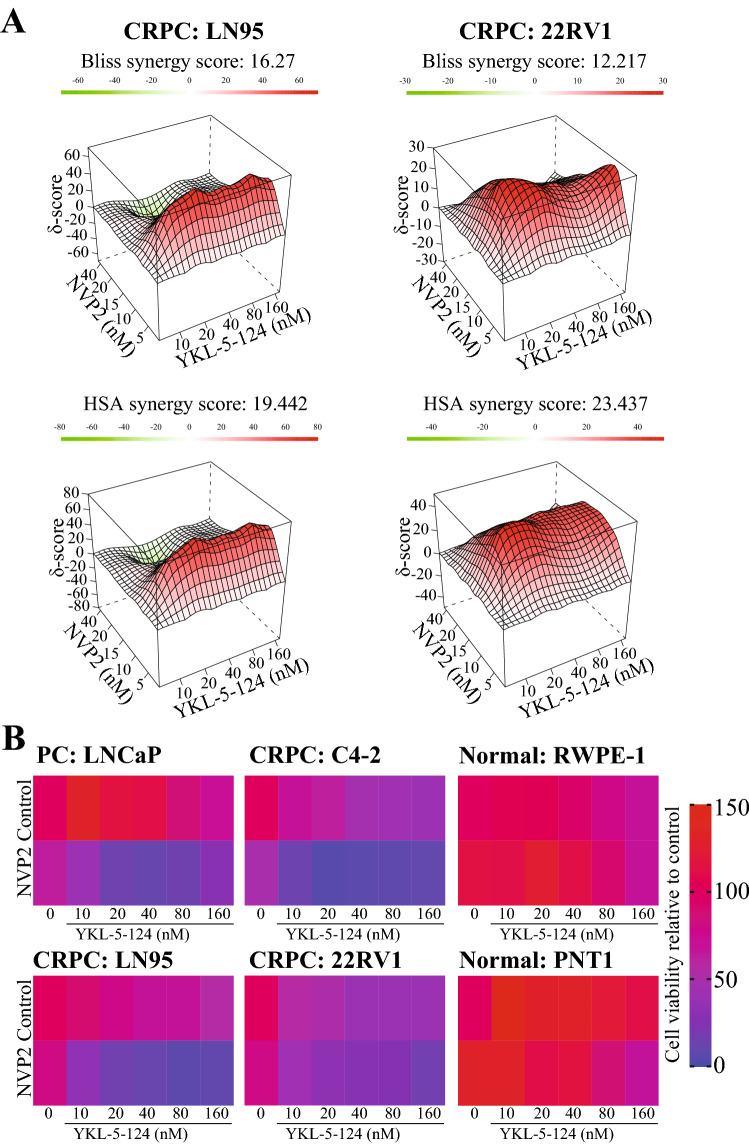
CDK7 inhibition causes CRPC-selective addiction on the high activity of transcription elongation kinase CDK9. **A** 3D synergy maps of BLISS and HSA scores between NVP2 and YKL-5-124 (two biological replicates). Cells were treated for 4 days after which cell viability was assessed using CellTiterGlo. Value higher than 10 indicates clear synergistic effects (Ianevski et al. 2020). **B** Cells were treated as indicated for 4 days and cell viability was assessed using CellTiterGlo (n: 2). NVP2 dose: 5 nM

CDK9 is directly druggable using both highly specific experimental compounds and drugs assessed in clinical trials, which enabled us to co-target CDK7 and CDK9. CDK9 activity can be depleted using the specific inhibitor NVP2 (Olson et al. 2018), which we have shown to be effective in low nanomolar doses against prostate cancer cells (Gondane et al. 2022a, b; Hu et al. 2021). Co-targeting of CDK7 and CDK9 synergistically blocked the proliferation of two CRPC models (Fig. 4A). We confirmed these effects using Samuraciclib, a CDK7 inhibitor currently evaluated in clinical trials and AT7519, a compound targeting CDK9 and previously assessed in clinical trials (Mahadevan et al. 2011; Chen et al. 2014) (Suppl. Figure 3).

CDK7 and CDK9 regulate fundamentally important steps in transcription, and it is therefore important to evaluate if their combinatorial inhibition is toxic to normal cells. We treated a panel of androgen-dependent prostate cancer, CRPC and normal prostate cells with a low dose of the CDK9 inhibitor NVP2 and increasing doses of the CDK7 inhibitor YKL-5-124. Strikingly, the combined inhibition of CDK7 and CDK9 led to an over 80% decrease in proliferation in the prostate cancer and CRPC cells but did not affect the cell lines derived from normal prostate epithelia (Fig. 4B). Based on these data, both prostate cancer and CRPC cells can be sensitized to CDK7 inhibitors by targeting CDK9. In contrast, these same CDK7 and CDK9 inhibitor doses are not toxic to normal prostate cells.

To summarize, our data establish CDK7 as a viable target for anti-CRPC therapy, and we also identify a candidate combinatorial treatment strategy.

## Discussion

In this work, we have shown that high CDK7 activity is important for the rapidly proliferating CRPC cells. First, we show that CDK7 and its positive regulators are overexpressed in aggressive prostate cancer (Fig. 1A, B). Next, we show that the CRPC cells, but not androgen-dependent prostate cancer cells, are sensitive to compounds targeting CDK7 (Fig. 1C–E). These data propose that CDK7 inhibitors could be combined with the mainstream prostate cancer therapy, anti-androgens: this treatment strategy should target both the androgen-dependent prostate cancer cells and the CRPC cells but have minimal effects on the normal cells of the body.

It was unexpected to us that CDK7 inhibitors selectively halt the proliferation of the CRPC cells and have limited effects on the normal cells. CDK7-dependent phosphorylation of RNA Pol II on serine 5 has been proposed as an essential step in the transcription initiation (Chou et al. 2020; Fisher 2019). However, the data generated by the DepMap-project show that practically all cell types tolerate siRNA-mediated decrease in CDK7-levels (Suppl. Figure 4). These data show that most cell types survive the lowered CDK7 activity, and imply that the current model of the role of CDK7 in transcription is incomplete. In the future, the precise role of CDK7 in transcription can be established using the highly specific inhibitors, YKL-5-124 (Olson et al. 2019) and Samuraciclib (Patel et al. 2018).

The CRPC-selective anomalies that initially conferred the growth advantage to cancer cells may explain why these cells are addicted on the high CDK7 activity. We show that co-targeting of CDK7 and OGT selectively blocks the proliferation of the CRPC cells (Fig. 2). Previously, it has been established that the selective CDK7 inhibitor YKL-5-124 predominantly affects the E2F transcription factor-driven gene expression (Olson et al. 2019). CRPC cells typically exhibit significantly higher activity of the E2F transcription factors due to the frequent inactivation of the negative regulator of the E2F-system, Retinoblastoma 1 (*RB1*) (Sharma et al. 2010). CDK7 inhibition should therefore affect a larger proportion of mRNAs in the *RB1* null cells than in the wild-type cells, and these effects should be dissected in more detail in the future studies. Another characterizing feature of the CRPC cells is inactivation of the *TP53* tumor suppressor gene, which allows rapid progression through cell cycle despite the existing DNA damage/incompletely replicated genome (Mateo et al. 2020). CDK7 has well-established role as the CDK-activating kinase, and it is possible that the loss of *TP53* renders the affected cells more reliant on the high CDK7 activity. In addition, and of a particular interest here, TP53 heterozygotic deletion often results in the loss of a nearby gene encoding for the catalytic subunit of RNA pol II (*POLR2A*), and the loss of *POLR2A* causes increased sensitivity to compounds targeting global transcription (Li et al. 2018). The existing literature and the data presented here suggest that CRPC cells are sensitive to CDK7 inhibitors due to the effects on both transcription and cell cycle.

Here, we show that the CRPC cells are addicted on the high activity of CDK7, and that depletion of CDK7 activity imposes stress to the transcription elongation machinery. In the future, it is important to establish what are the genes dependent on the high CDK7 activity, and how OGT affects transcription when CDK7 is compromised.

## Supplementary Information

Below is the link to the electronic supplementary material.

Supplementary file1 (PDF 935 KB)

Supplementary file2 (XLSX 15 KB)
