# Supplementary material for: Castration-resistant prostate cancer cells are dependent on the high activity of CDK7
Source: J Cancer Res Clin Oncol. 2022 Nov 18;149(8):5255–63. doi: 10.1007/s00432-022-04475-3 (PMC10349716; doi:10.1007/s00432-022-04475-3)
Supplement: Supplementary file 1 — Supplementary file1 (PDF 935 KB) [file 432_2022_4475_MOESM1_ESM.pdf]

Supplementary figures and supplementary figure legends

Supplementary Table 1. Proteins increasingly O-GlcNAcylated in response to CDK7 inhibitor treatment. Cells were treated for 4 hours with either DMSO (vehicle control) or 500nM YKL-5-124, O-GlcNAcylated proteins immunoprecipitated and identified using mass spectrometry. Data shown is an average of three biological replicates in each condition. The signal intensity values represented here were normalized to IgG. O-GlcNAcylation of each of these proteins was increased at least by 20% in each of the biological replicates.

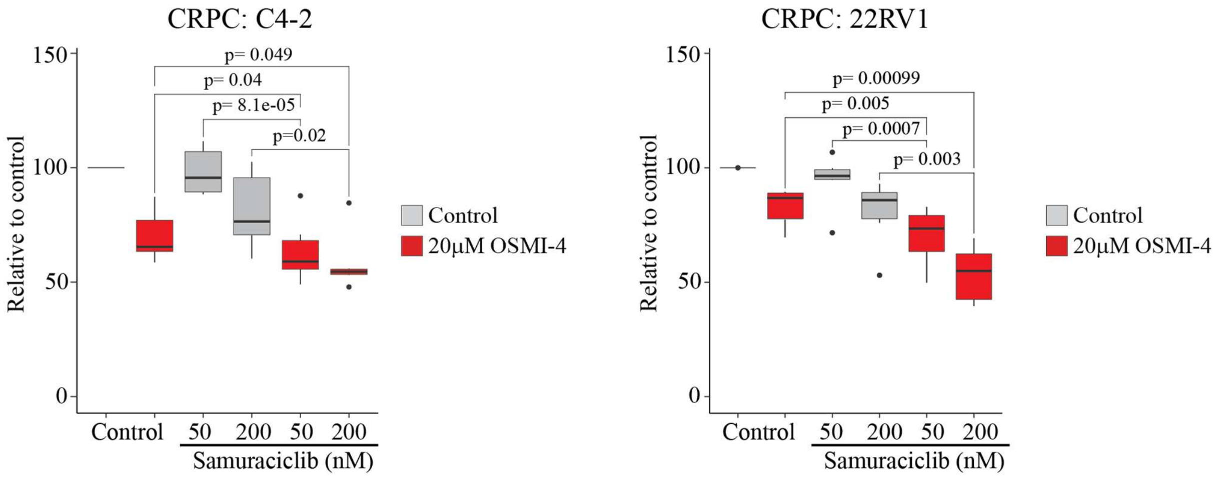

Supplementary figure 1. OGT inhibition enhances the anti-proliferative effects of CDK7 inhibitor Samuraciclib. Data shown is from five biological replicates and two-tailed, paired samples Student's t-test was used to evaluate the significance. Control sample was always set to value of 100, and the rest of the samples are presented relative to this.

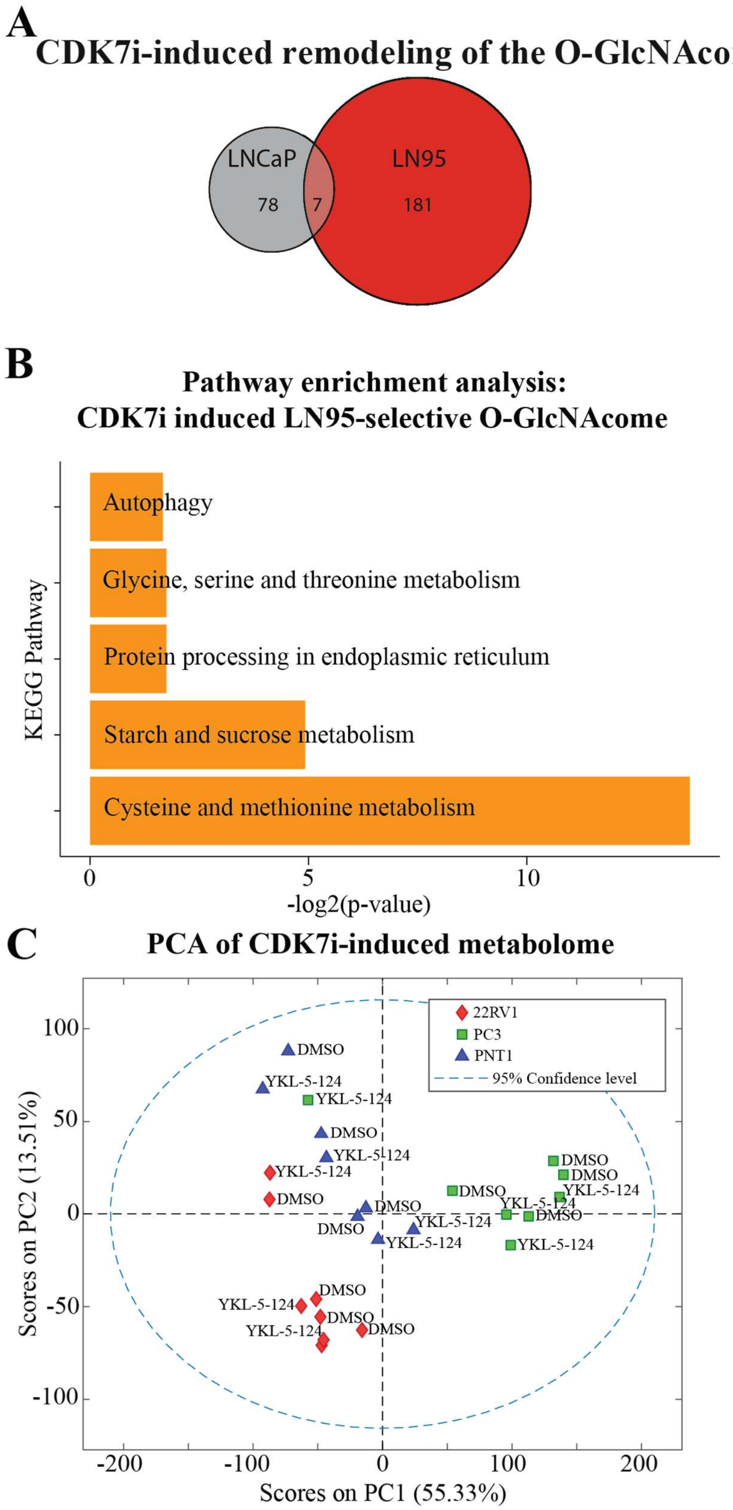

Supplementary figure 2. CDK7 inhibitor treatment and mass spectrometry-based detection of O-GlcNAcylated proteins. A) Proteins whose O-GlcNAcylation increases by at least 20% in all three biological replicates. Summary of the mass spectrometry data. **B)** KEGG pathway enrichment analysis of the proteins whose O-GlcNAcylation increased by at least 20% in all biological replicates in CRPC cells but not in prostate cancer cells. **C)** Principal component analysis (PCA) of the NMR-based metabolite profiling after the indicated treatments. Cells were treated with either control (DMSO) or 50nM YKL-5-124 for 24 hours.

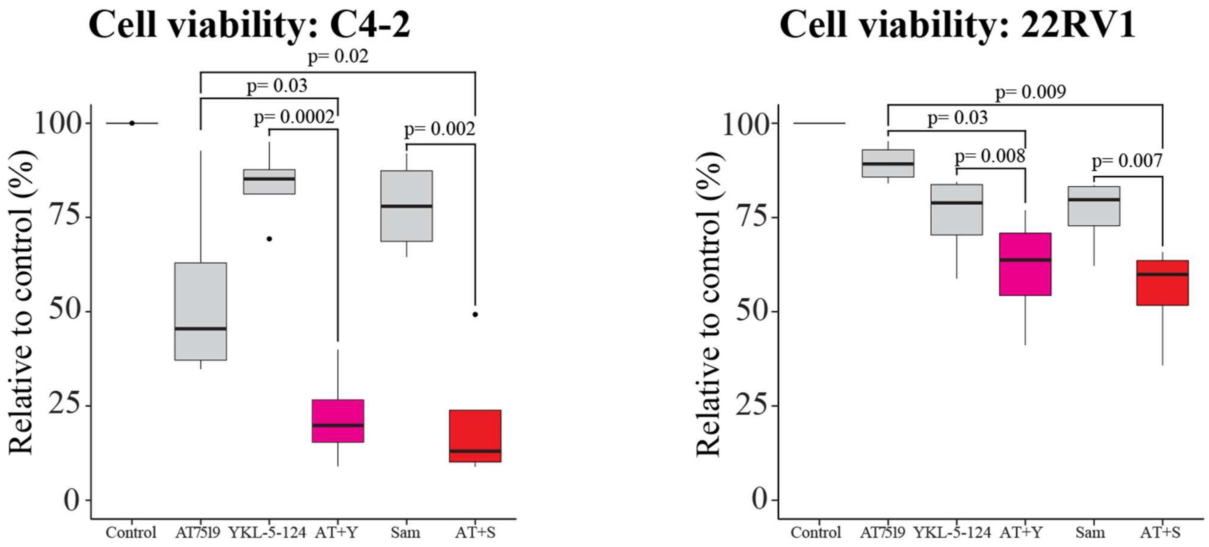

Supplementary figure 3. Targeting CDK9 sensitizes CRPC cells to CDK7 inhibition. Data shown is from four biological replicates and two-tailed, paired samples Student's t-test was used to evaluate the significance. Control sample was always set to value of 100, and the rest of the samples are presented relative to this. AT7519: 0.5µM, YKL-5-124: 20nM and Samuraciclib: 200nM.

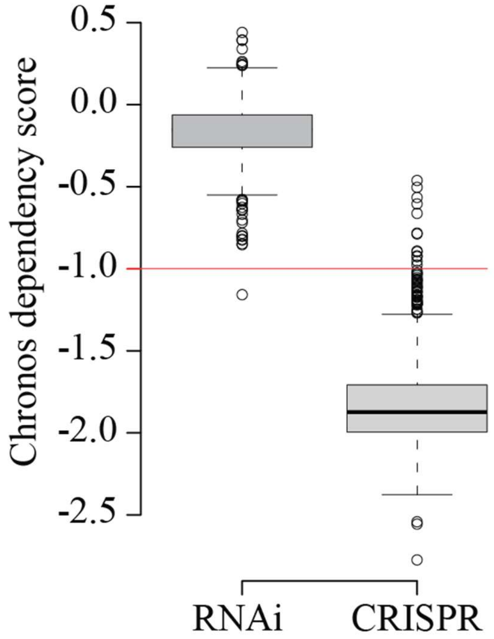

Supplementary figure 4. Most cells survive with low levels of CDK7. Knockdown of CDK7 has minimal effects on most cell types, while knockout using CRISPR is lethal to most cell types. The plot was generated using the data available through the DepMap database (DepMap 21Q4 Public+Score, Chronos) (Meyers et al. 2017). The score of 0 indicates that the gene is non-essential, and score of -1 (highlighted in red here) is the median of all pan-essential genes.

References

Meyers, R. M., J. G. Bryan, J. M. McFarland, B. A. Weir, A. E. Sizemore, H. Xu, N. V. Dharia, P. G. Montgomery, G. S. Cowley, S. Pantel, A. Goodale, Y. Lee, L. D. Ali, G. Jiang, R. Lubonja, W. F. Harrington, M. Strickland, T. Wu, D. C. Hawes, V. A. Zhivich, M. R. Wyatt, Z. Kalani, J. J. Chang, M. Okamoto, K. Stegmaier, T. R. Golub, J. S. Boehm, F. Vazquez, D. E. Root, W. C. Hahn, and A. Tsherniak. 2017. 'Computational correction of copy number effect improves specificity of CRISPR-Cas9 essentiality screens in cancer cells', *Nat Genet*, 49: 1779-84.
